# Depression in Adolescence: Relevance of Serotonin Receptor Polymorphisms

**DOI:** 10.1155/da/5239931

**Published:** 2025-05-19

**Authors:** Giulia Gizzi, Federico Fiorani, Elisabetta Albi, Samuela Cataldi, Claudia Mazzeschi, Elisa Delvecchio

**Affiliations:** ^1^Department of Philosophy, Social Sciences and Education, University of Perugia, Perugia, Italy; ^2^Department of Pharmaceutical Sciences, University of Perugia, Perugia 06123, Italy

**Keywords:** 5-HT2A receptor, adolescents, depression symptoms, rs6311 polymorphism, rs6313 polymorphism

## Abstract

Depression in adolescence is influenced by the environment, family members, social relationships, and genetic factors. Gene polymorphisms of serotonin receptors were associated with mental/psychiatric disorders, including impulsive, aggressive, violent, antisocial or criminal conduct, schizophrenia, eating disorders, alexithymia, autism, and major depressive disorder (MDD). Few studies reported the association between serotonin receptor polymorphisms and depressive symptoms in nonclinical subjects. The present study aimed to evaluate the serotonin receptor polymorphisms in nonclinical adolescents presenting depressive symptoms. The results clearly demonstrated that nonclinical adolescents with severe depressive symptoms had a high concentration of GA + AA in the recessive model and of AA in the homozygous model of the rs6311 polymorphism. The data indicated that the A allele was associated with severe depressive symptoms. Moreover, the analysis highlighted a trend of association of TT + CT in the recessive model for rs6313 polymorphism and severe symptoms. In conclusion, our study confirms that the A allele for rs6311 represents a risk factor, and the allele T for rs6313 could be a possible risk factor for severe depressive symptoms. As a consequence, the allele G for rs6311 and the allele C for rs6313 could be protective against severe depressive symptoms. Therefore, it might be appropriate to work preventively on adolescents with the A allele for rs6311 and T allele for rs6313 polymorphism to reduce the possibility of developing depressive symptoms and to preserve mental health in young people.

## 1. Introduction

Adolescence is a phase of life full of challenges and changes in which the subject prepares to take on the role and identity of the adult from a physical, cognitive, social, and emotional point of view [1]. Poor emotion regulation was associated with externalizing problems, and excessive inhibition in emotion regulation was related to internalizing problems, such as anxious and depressive symptoms [2]. To date, several lines of evidence have demonstrated that abnormalities in the function of the central serotonergic system were implicated in the pathogenesis of depressive illness. Among serotonin or 5-hydroxytryptamine 5-HT receptors, 5-HT2A had the highest concentration in the brain with particular density in the cortex, amygdala, hippocampus, and basal ganglia [3]. 5-HT2A receptor has been genetically and functionally associated with various neuropsychiatric disorders [4–6]. The 5-HT2A gene was considered a target of various antidepressant treatments [7].

The most studied single-nucleotide polymorphisms (SNPs) of 5-HT2A receptor gene are: (1) a variant close to the promoter region (−1438A/G) or rs6311 SNP and (2) a silent variant in exon 1 (102T/C) or rs6313 SNP. The two polymorphisms were reported in complete linkage disequilibrium. The high-activity mutant A allele of HTR2A rs6311 polymorphism corresponded to the T allele of HTR2A-rs6313 polymorphism [8]. The concept of gene variations for understanding brain pathology has evolved over the past several decades and continues to gain considerable traction. A strong association between rs6311-rs6313 and psychiatric disorders has been demonstrated [9–11]. Studies conducted in groups of different ethnic groups (Germany, Japan, Africa, and Russia) have highlighted that the subjects GG or AG for the rs6311 variant are more impulsive, aggressive, violent, and more direct to antisocial or criminal conduct [12]. Moreover, in patients affected by schizophrenia, the frequencies of GG (30%, rs6311) and CC genotypes (32%, rs6313) were higher than in controls [13]. GG genotype (rs6311) was also associated with increased risk for suicide as a comorbidity of eating disorders [14]. Again, the G allele (rs6311) was related to alexithymia compared to the AA genotype [15]. Children with autism spectrum disorder carrying the GG genotype of rs6311 had more severe symptoms of depression than subjects with genotypes AA or GA [16]. Li et al. [17] demonstrated that adolescent females aged 12–15 years with the CC genotype (rs6313) showed more depressive symptoms when exposed to high levels of peer victimization and fewer depressive symptoms when peer victimization was lower. Eley et al. [18] showed that in adolescents aged 12–19 years, the T allele (rs6313) made them more vulnerable to high depressive symptoms. The work aimed to evaluate the association between polymorphisms of the 5-HTR2A (rs6311 and rs6313) and different levels of depressive symptoms (absent to sporadic, mild, moderate, and severe) in a well-selected population of nonclinical adolescents (14–17 years, Umbria, Italy).

## 2. Methods

### 2.1. Participants

One hundred and fifty adolescents attending secondary schools in Umbria (Italy) were recruited in this study. The adolescents were aged between 14 and 17 years, with a mean age of 15.13 (SD = .99) (Table 1). 67.3% (*n* = 101) of the adolescents were female and 32.7% (*n* = 49) were male. The sample was homogeneously distributed by sex and age (*χ*^2^ = 7.729, *p*=0.05). The inclusion criteria included: nonclinical adolescents, Italian language, informed consent signed by both parents, and the researchers had no family or friendship ties (for ethical reasons and to avoid invalidating the textological evidence). <5% of the study population reported having consulted a psychologist for emotional problems in the past 2 years. The exclusion criteria were students older than those indicated as repeating students of the school year and diagnosed with a mood disorder. The project was approved by the Bioethics Committee of the University of Perugia (n.69574 approved on April 13, 2021) and all procedures were performed accordingly. Participation in the study was voluntary and parents signed informed consent for participation. Participants were anonymized and no sensitive data were collected.

### 2.2. Assessment of Depressive Symptoms

For the assessment of depressive symptoms, the DSM-5 Level 2 Cross-Symptom Rating Scale was used (PROMIS Emotional Distress–Depression Pediatric Item Bank; American Psychiatric Association, 2013), a self-report questionnaire assessing depressive symptoms in adolescents aged 11–17 years, consisting of 14 items, the scores of which are rated on a 5-point Likert scale from 1 = never to 5 = almost always. Specifically, the questionnaire investigates how often in the last 7 days the subject experienced the different symptoms. An example of an item is “Being sad has made it difficult for me to do things with my friends”. Four cut-offs were given by the American Psychological Association (APA) to indicate the levels of depressive symptoms: (1) less than 55 = absent to sporadic, (2) 55.0–59.9 = mild, (3) 60.0–69.9 = moderate, and (4) 70 and more = severe. Cronbach's Alpha is 0.94 (95% CI = [0.93, 0.96]) for the study population.

### 2.3. Collection of Saliva Samples

All participants followed the same protocol for saliva sample collection using specific disposable sterile swabs, and each provided a saliva sample after a morning fast. The samples were stored at −80°C.

### 2.4. Genomic DNA Extraction

Genomic DNA was isolated from saliva samples by using the Monarch Genomic DNA Purification Kit (New England Biolabs, Massachusetts, US) as previously reported [19].

### 2.5. Genotyping Analysis

rs6311 and rs6313, as the most studied SNPs of 5-HTR2A (see Introduction), were analyzed. rs6311 (ID: C___8695278_10) and rs6313 (ID: C___3042197_1) genotyping was performed by the StepOne 7500 Real-Time PCR system using TaqMan SNP Genotyping Assays (Thermo Fisher Scientific, Waltham, MA). The genotyping procedure was performed as previously reported [19].

### 2.6. Statistical Analyses

Descriptive statistics were used to examine genotype distributions and test for Hardy–Weinberg equilibrium. Linkage disequilibrium between rs6311 and rs6313 was also assessed. Associations between genotypes and depressive symptom severity were analyzed using chi-square tests (*χ*^2^), with Cramér's V estimating effect sizes. Equivalence tests (odds ratios and confidence intervals) assessed whether differences were practically meaningful [15]. To further explore these relationships, dominant, recessive, and homozygous genetic models were tested. Post hoc analyses included Fisher's Exact Test, the G-test, and adjusted residual analysis [20, 21], with Bonferroni corrections for multiple comparisons. A significance threshold of *p*  < 0.05 was applied, with adjusted *p*-values (*p*_Adj_) reported for post hoc tests. Analyses were performed using SPSS 26 for general statistics and R 4.4.3 for advanced modeling and equivalence testing.

## 3. Results

To establish the relationship between the rs6311 and rs6313 polymorphisms of the 5-HT2A receptor gene and depressive symptoms in nonclinical adolescents, we first investigated the allelic dominance and distribution of the two genotypes, known to be in linkage disequilibrium, in the study population. The results showed that the major allele for rs6311 was G (*n* = 117; 53,9%), while for rs6313 it was C (*n* = 113, 50,9%). Consequently, the minor allele for rs6311 was A (*n* = 100, 46,1%) and for rs6313 T (*n* = 109, 49,1%). The distribution was checked with Hardy–Weinberg formula, with the same results.

The two polymorphisms were almost in complete linkage disequilibrium (the A and G alleles of rs6311 correspond respectively to the T and C alleles of rs6313). Only ten subjects (6.6%) presented a different allelic distribution (five samples of genotype GG correspond to CT; three samples of genotype GG correspond to TT; one sample of genotype GA corresponds to TT; one sample of genotype AA corresponds to CT).

Furthermore, the results showed that in the study population, the GG homozygote was 1.5-fold higher than the AA for rs6311 and the CC homozygote was 1.1-fold higher than the TT for rs6313. The GA heterozygote for rs6311 was 7% lower than the CT heterozygote for rs6313 (Table 2).

Thus, we evaluated whether there was a difference in the genotype frequency of the rs6311 and rs6313 polymorphisms in different levels of depressive symptoms. The analysis did not reveal statistically significant differences in genotype frequencies of rs6311 and rs6313 across different levels of depressive symptoms. However, effect size estimates and equivalence tests suggested meaningful variation in symptom distribution among genotypes.

For rs6311 (Table 3), the chi-square test (*χ*^2^ = 12.182, *p*=0.06) indicated a weak trend toward an association (Cramér's V = 0.2015), though not statistically significant. However, the equivalence test using odds ratios suggested that genotype differences were non-negligible (OR = 0.3, 95% CI: 0.2189–0.3958), as the confidence interval fell outside the predefined equivalence range (0.80–1.25). Post hoc analyses identified significant differences, with the GG genotype showing an absence of severe symptoms, while AA was associated with a higher prevalence of severe cases (*p*_Adj_ = 0.02). GA displayed an intermediate profile with more moderate symptoms.

For rs6313 (Table 3), the chi-square test (*χ*^2^ = 9.661, *p*=0.14) did not indicate a significant association, though a small effect size (Cramér's V = 0.179) suggested a weak trend. The equivalence test confirmed that observed differences were non-negligible, as ORs for CT (1.97, 95% CI: 0.71–5.93) and TT (1.32, 95% CI: 0.38–4.64) exceeded the equivalence range. Post hoc analyses revealed that CC significantly differed from both CT and TT, with CT showing a higher prevalence of moderate symptoms and TT displaying a more balanced severity distribution (*p*_Adj_ = 0.05). No significant differences emerged between CT and TT, suggesting a similar symptom distribution. Overall, while no statistically significant associations were detected, equivalence tests and effect size estimates suggest that rs6311 and rs6313 genotypes may influence the severity distribution of depressive symptoms.

Subsequently, to better investigate the relationship between rs6311 and rs6313 polymorphisms and depressive symptoms severity, dominant, recessive, and homozygous genetic models were used [22].

### 3.1. Genetic Models of rs6311 Polymorphism and Depressive Symptoms

No significant association emerged in the dominant model (*χ*^2^ = 5.772, *p*=0.12, Cramér's V = 0.196), and equivalence testing suggested that differences in symptom prevalence were not practically meaningful. The recessive model (*χ*^2^ = 8.376, *p*=0.04, Cramér's V = 0.236) and the homozygous model (*χ*^2^ = 9.940, *p*=0.02, Cramér's V = 0.346) indicated significant associations, suggesting that genotype distribution influences symptom severity (Table 4). Specifically, in the recessive model, GG was associated with a lower prevalence of severe symptoms compared to GA + AA (*p*_Adj_ = 0.02), while in the homozygous model, AA showed a significantly higher prevalence of severe symptoms than GG (*p*_Adj_ = 0.007). Equivalence tests confirmed that these differences exceeded the predefined equivalence threshold (±10%), reinforcing the hypothesis of a potential protective effect of the GG genotype against severe depressive symptoms.

### 3.2. Genetic Models of rs6313 Polymorphism and Depressive Symptoms

No statistically significant associations were found in the dominant (*χ*^2^ = 4.387, *p*=0.22), recessive (*χ*^2^ = 6.765, *p*=0.08), or homozygous (*χ*^2^ = 7.672, *p*=0.05) models, though effect size estimates suggested potential weak-to-moderate relationships. Equivalence tests revealed that differences in symptom prevalence exceeded the predefined range, particularly in the recessive and homozygous models, suggesting non-negligible variations despite the lack of strong statistical significance. Post hoc analyses indicated that CC had a lower prevalence of severe symptoms compared to CT + TT (*p*_Adj_ = 0.02), and CC differed significantly from TT (*p*_Adj_ = 0.02) in the homozygous model, reinforcing a potential genotype-dependent effect on symptom distribution.

## 4. Discussion

Correlation between the rs6311 and rs6313 5-HT2A polymorphisms and different types of pathologies was reported. Interestingly, with respect to rs6311 polymorphism, carriers of the A allele tended to suffer from major depressive disorder (MDD) [23]. The AA genotype was associated with higher depressive symptoms in association with low cultural consonance than the GA or GG genotypes [24]. A recent meta-analysis showed that in nonclinical subjects from the Caucasian population, the G allele is the major allele for rs6311 and the C allele is the major allele for rs6313 [25]. In agreement with these data, our results showed equal allelic dominance in Umbrian nonclinical adolescents. Of note, our results showed a lower percentage of the GG and GA allele pairs and a higher percentage of the AA pair. About rs6313, our results showed a smaller percentage of the CC allele pair. It is possible that, despite being a nonclinical sample, our population did not have the same distribution of genotypes as it included subjects with mild, moderate, and severe depressive symptoms. In fact, the AA genotype (rs6311) has been associated with increased vulnerability to depressive symptoms [23]. We had hypothesized that in our sample, there would be a prevalence of the AA genotype (rs6311) and the TT genotype (rs6313) in relation to high levels of depressive symptoms, as previously suggested [18, 24]. However, investigating the simple distribution of genotypes in relation to the different levels of depressive symptoms, no statistically significant difference was found. However, equivalence testing resulted in a trend of association with severe symptoms, whether the genotype was AA, and with moderate symptoms, whether the genotype was GA, for the rs6311 polymorphism. Moreover, subjects with TT and CT for rs6313 polymorphism showed a different trend with respect to CC. Of note, TT had the balanced distribution with respect to the gravity of the symptoms and CT had a trend of association with moderate symptoms. Then we analyzed the genetic models considering that a simple distribution of genotypes is used as the first step to obtain a starting overview. We showed that subjects with severe levels of depressive symptoms presented a higher concentration of GA + AA in the recessive model and of AA in the homozygous model of rs6311 polymorphism. These data explain the higher percentages of AA within our sample compared to the Caucasian population [25]. Interestingly, carriers of the A allele of the rs6311 polymorphism tended to suffer from MDD [24].

Since the two polymorphisms rs6311 and rs6313 are in almost complete linkage disequilibrium, an association between CC+TT in the recessive model and TT in the homozygous model of rs6113 with depressive symptoms could also be expected. In reality, the values obtained are very close to significance. The small percentage of subjects who do not have linkage disequilibrium influences the outcome. The equivalence testing confirmed the association of the AA + GA recessive model with severe symptoms. Moreover, this analysis highlighted a trend of association of the recessive model TT + CT and severe symptoms for rs6313. In conclusion, our study confirms that the A allele for rs6311 represents a risk factor and the allele T for rs6313 could be a possible risk factor for severe depressive symptoms. As a consequence, the allele G for rs6311, and the allele C for rs6313 could be protective against severe depressive symptoms. Future studies on a wider population could be useful to confirm these data.

## Figures and Tables

**Table 1:** The distribution of sex by age.

Total subjects	Total (*N* = 150)		14 years old (*n* = 55)		15 years old (*n* = 29)		16 years old (*n* = 57)		17 years old (*n* = 9)	
	*N*	*%*	*n*	*%*	*n*	*%*	*n*	*%*	*n*	*%*
Boys	49	32.7	11	20.0	10	34.5	28	42.4	5	55.6
Girls	101	67.3	44	80.0	19	65.5	38	57.6	4	44.4

**Table 2:** Genotype distribution of rs6311 and rs6313polymorphisms (*N* = 150).

Genotypes	rs6311	
	*n*	%
GG	50	33.3
GA	67	44.7
AA	33	22.0

**Genotypes**	**rs6313**	
	***n***	**%**

CC	41	27.3
CT	72	48.0
TT	37	24.6

**Table 3:** Frequencies and chi-square tests for rs6311 and rs6313 genotype distribution in different levels of depressive symptoms (*N* = 150).

Depressive symptoms	rs6311 genotypes								*χ* ^2^
	GG		GA		AA		Total		
	*n*	%	*n*	%	*n*	%	*n*	%	
Absent to sporadic	29	58	26	38.8	16	48.5	71	47.3	
Mild	10	20	15	22.4	6	18.2	31	20.7	
Moderate	11	22	19	28.4	5	15.2	35	22.3	
Severe	0	0	7	10.4	6	18.2	13	8.7	
Total	50	100	67	100	33	100	150	100	12.182

**Depressive symptoms**	**rs6313 genotypes**								***χ* ^2^**
	**CC**		**CT**		**TT**		**Total**		
	***n***	**%**	***n***	**%**	***n***	**%**	***n***	**%**	

Absent to sporadic	24	58.5	29	40.3	18	48.6	71	47.2	
Mild	7	17.1	17	23.6	7	18.9	31	20.7	
Moderate	10	24.4	19	26.4	6	16.2	35	23.3	
Severe	0	0	7	9.7	6	16.2	13	8.7	
Total	41	100	72	100	37	100	150	100	9.661

*⁣* ^*∗*^ *p* < 0.05.

**Table 4:** Frequencies and chi-square tests for recessive pattern and for homozygosity pattern of the rs6311 polymorphism in different levels of depressive symptoms (*N* = 150).

Depressive symptoms	rs6311-recessive model								*χ* ^2^
	GA + AA			GG			Total		
	*n*	%	Adjusted residual	*n*	%	Adjusted residual	*n*	%	
Absent to sporadic	42	42	−1.9	29	58	1.9	71	47.3	—
Mild	21	21	0.1	10	20	−0.1	31	20.7	—
Moderate	24	24	0.3	11	22	−0.3	35	23.3	—
Severe	13	13	2.7*⁣*^*∗∗*^	0	0	−2.7	13	8.7	—
Total	100	100	—	50	100	—	150	100	8.376*⁣*^*∗*^

**Depressive symptoms**	**rs6311-homozygous model**								***χ* ^2^**
	**GG**			**AA**			**Total**		
	***n***	**%**	**Adjuster residual**	***n***	**%**	**Adjuster residual**	***n***	**%**	

Absent to sporadic	29	58	0.9	16	48.5	−0.9	45	54.2	—
Mild	10	20	0.2	6	18.2	−0.2	16	19.3	—
Moderate	11	22	0.8	5	15.2	−0.8	16	19.3	—
Severe	0	0	−3.1	6	18.2	3.1*⁣*^*∗∗*^	6	7.2	—
Total	50	100	—	33	100	—	83	100	9.940*⁣*^*∗*^

*⁣* ^*∗*^ *p* < 0.05; *⁣*^*∗∗*^*p* < 0.01.

## Data Availability

All data generated and/or analyzed during the current study are in the paper.

## References

[1] Andrews J. L., Foulkes L., Blakemore S. J. (2020). Peer Influence in Adolescence: Public-Health Implications for COVID-19. *Trends in Cognitive Sciences*.

[2] Rydell A., Thorell L. B., Bohlin G. (2007). Emotional Regulation in Relation to Social Functioning: An Investigation of Child Self-Report. *European Journal of Developmental Psychology*.

[3] Voronova I. P. (2021). 5-HT Receptors and Temperature Homeostasis. *Biomolecules*.

[4] Naughton M., Mulrooney J. B., Leonard B. E. (2000). A Review of the Role of Serotonin Receptors in Psychiatric Disorders. *Human Psychopharmacology: Clinical and Experimental*.

[5] Dean B., Hayes W. (1996). Decreased Frontal Cortical Serotonin2A Receptors in Schizophrenia. *Schizophrenia Research*.

[6] Rodríguez J. J., Noristani H. N., Verkhratsky A. (2012). The Serotonergic System in Ageing and Alzheimer’s Disease. *Progress in Neurobiology*.

[7] Choi M. J., Kang R. H., Ham B. J., Jeong H. Y., Lee M. S. (2005). Serotonin Receptor 2A Gene Polymorphism (-1438A/G) and Short-Term Treatment Response to Citalopram. *Neuropsychobiology*.

[8] Lencz T., Malhotra A. K., Cohen N. (2008). Pharmacogenomics Applications in Psychiatric Disorders. *Pharmacogenomics and Personalized Medicine*.

[9] Massoud S., Salmanian M., Tabibian M., Ghamari R., Tavabe Ghavami T. S., Alizadeh F. (2023). The Contribution of the 5-Hydroxytryptamine Receptor 2 A Gene Polymorphisms rs6311 and rs6313 to Schizophrenia in Iran. *Molecular Biology Reports*.

[10] Li L., Yang Y., Zhang Q., Wang J., Jiang J., Neuroimaging Initiative AD (2021). Use of Deep-Learning Genomics to Discriminate Healthy Individuals From Those With Alzheimer’s Disease or Mild Cognitive Impairment. *Behavioural Neurology*.

[11] Du D., Tang Q., Han Q. (2000). Association between Genetic Polymorphism and Antidepressants in Major Depression: A Network Meta-Analysis. *Pharmacogenomics*.

[12] Nedic Erjavec G., Tudor L., Nikolac Perkovic M. (2022). Serotonin 5-HT2A Receptor Polymorphisms are Associated With Irritability and Aggression in Conduct Disorder. *Progress in Neuro-Psychopharmacology and Biological Psychiatry*.

[13] Sujitha S. P., Nair A., Banerjee M. (2014). 5-Hydroxytryptamine (Serotonin) 2A Receptor Gene Polymorphism Is Associated With Schizophrenia. *The Indian Journal of Medical Research*.

[14] Genis-Mendoza A. D., Ruiz-Ramos D., López-Narvaez M. L. (2019). Genetic Association Analysis of 5-HTR2A Gene Variants in Eating Disorders in a Mexican Population. *Brain and Behavior*.

[15] Hoenig J. M., Heisey D. M. (2001). The Abuse of Power: The Pervasive Fallacy of Power Calculations for Data Analysis. *The American Statistician*.

[16] Gadow K. D., Smith R. M., Pinsonneault J. K. (2014). Serotonin 2a Receptor Gene (Htr2a) Regulatory Variants: Possible Association With Severity of Depression Symptoms in Children With Autism Spectrum Disorder. *Cognitive and Behavioral Neurology*.

[17] Li Y., Ma X., Feng C., Wang Y. (2022). Parental Psychological Control and Adolescents Depression During the COVID-19 Pandemic: The Mediating and Moderating Effect of Self-Concept Clarity and Mindfulness. *Current Psychology*.

[18] Eley T. C., Sugden K., Corsico A. (2004). Gene-Environment Interaction Analysis of Serotonin System Markers With Adolescent Depression. *Molecular Psychiatry*.

[19] Ceccarini M. R., Fittipaldi S., Ciccacci C. (2022). Association Between DRD2 and DRD4 Polymorphic Variant and Eating Disorders in an Italian Population. *Frontiers in Nutrition*.

[20] Beasley T. M., Schumacker R. E. (1995). Multiple Regression Approach to Analyzing Contingency Tables: Post Hoc and Planned Comparison Procedures. *The Journal of Experimental Education*.

[21] García-pérez M. A., Núñez-antón V. (2003). Cellwise Residual Analysis in Two-Way Contingency Tables. *Educational and Psychological Measurement*.

[22] Lin J.-Y., Jiang M.-Y., Kan Z.-M., Chu Y. (2014). Influence of 5-HTR2A Genetic Polymorphisms on the Efficacy of Antidepressants in the Treatment of Major Depressive Disorder: A Meta-Analysis. *Journal of Affective Disorders*.

[23] Zhao X., Sun L., Sun Y. H. (2014). Association of HTR2A T102C and A-1438G Polymorphisms With Susceptibility to Major Depressive Disorder: A Meta-Analysis. *Neurological Sciences*.

[24] Dressler W. W., Balieiro M. C., Ribeiro R. P., Dos Santos J. E. (2009). Cultural Consonance, a 5HT2A Receptor Polymorphism, and Depressive Symptoms: A Longitudinal Study of Gene x Culture Interaction in Urban Brazil. *American Journal of Human Biology: The Official Journal of the Human Biology Council*.

[25] White K. C., McDonald A. K., Compton D. M. (2022). 5-HTR2A Polymorphisms rs6311 and rs6313 and Major Depressive Disorder: A Meta-Analysis. *Journal of Behavioral and Brain Science*.

